# Pegfilgrastim and daily granulocyte colony-stimulating factor: patterns of use and neutropenia-related outcomes in cancer patients in Spain – results of the LEARN Study

**DOI:** 10.1111/j.1365-2354.2008.00959.x

**Published:** 2009-05

**Authors:** D ALMENAR, J MAYANS, O JUAN, JM GARCIA BUENO, JI JALON LOPEZ, A FRAU, M GUINOT, P CEREZUELA, E GARCIA BUSCALLA, JA GASQUET, J SANCHEZ

**Affiliations:** 1 Department of Oncology, Hospital Universitario Dr PesetValencia; 2 Department of Hematology, Hospital Arnau de VilanovaValencia; 3 Department of Oncology, Hospital Arnau de VilanovaValencia; 4 Department of Oncology, Hospital General UniversitarioAlbacete; 5 Department of Oncology, Clínica RúberMadrid; 6 Department of Oncology, Hospital Provincial de CastellónCastellón; 7 Department of Hematology, Hospital General de CastellónCastellón; 8 Department of Oncology, Hospital Santa María del RosellCartagena; 9 Medical Department, Amgen S.A.Barcelona, Spain

**Keywords:** pegfilgrastim, G-CSF, pattern of use, neutropenia, febrile neutropenia

## Abstract

Daily granulocyte colony-stimulating factors [(G-CSFs); e.g. filgrastim, lenograstim] are frequently used to reduce the duration of chemotherapy-induced neutropenia (CIN) and the incidence of febrile neutropenia (FN) in cancer patients. A pegylated formulation of filgrastim, pegfilgrastim, which is administered once per cycle, was introduced in Spain in 2003. LEARN was a multi-centre, retrospective, observational study in Spain comparing patterns of use of daily G-CSF and pegfilgrastim, and CIN-related outcomes in adults with non-myeloid malignancies receiving myelosuppressive chemotherapy. Outcome measures were the percentage of patients receiving G-CSF for primary prophylaxis versus secondary prophylaxis/treatment, duration of treatment with G-CSF and incidence of CIN-related complications. Medical records from consecutive patients with documented pegfilgrastim (*n* = 75) or daily G-CSF (*n* = 111) use during 2003 were included. The proportion of patients receiving primary or secondary prophylaxis was comparable between the pegfilgrastim (39 and 48% respectively) and daily G-CSF (40 and 48% respectively) groups. However, there was a trend towards less frequent use to treat a neutropenic event such as FN or neutropenia in the pegfilgrastim group (17 versus 30% with daily G-CSF). Chemotherapy-induced neutropenia-related complications were less frequent in patients receiving pegfilgrastim (e.g. FN 11 versus 24% with daily G-CSF). This is the first study to show the potential benefits of pegfilgrastim over daily G-CSF in Spanish clinical practice.

## INTRODUCTION

Chemotherapy-induced neutropenia (CIN) is the most common dose-limiting toxicity of cancer chemotherapy. Patients receiving myelosuppressive chemotherapy frequently develop severe grade 3 or 4 neutropenia or febrile neutropenia (FN), which can make them susceptible to the development of potentially life-threatening infections (Dale *et al*. 2001; Aapro *et al*. 2006; Smith *et al*. 2006).

Historically, the principal strategies for managing CIN are reducing the dose intensity and total dose of the chemotherapy. In the case of FN, a medical emergency, hospitalization is generally required, with the administration of antibiotics to combat infection. It is, however, well documented that reducing or delaying the chemotherapy dose can compromise treatment outcomes and ultimately patient survival (Bonadonna *et al*. 1995; Budman *et al*. 1998). Moreover, use of antibiotics can be lifesaving but may be associated with adverse effects and the emergence of resistant pathogens (Yoshida & Ohno 2004).

The introduction of granulocyte colony-stimulating factors (G-CSFs) over a decade ago has had a significant impact on the management of myelotoxicity associated with cancer chemotherapy. Daily subcutaneous doses of G-CSFs such as filgrastim (Neupogen®, Amgen, Inc., Thousand Oaks, CA, USA) and lenograstim (Granocyte®, Chugai Sanofi-Aventis, Paris, France) are now commonly used in clinical practice to reduce the incidence, duration and severity of CIN, the incidence of FN, and the risk of infection in patients undergoing myelosuppressive chemotherapy (Crawford *et al*. 1991; Trillet-Lenoir *et al*. 1993).

Guidelines published by the National Comprehensive Cancer Network recommend the routine prophylactic use of CSFs to prevent the development of FN in patients in whom the risk of developing FN or a neutropenic event compromising treatment with systemic chemotherapy is 20% or higher (defined as ‘high-risk’ patients) (Crawford *et al*. 2005; Lyman 2005). Updated guidelines from the American Society for Clinical Oncology and from the European Organization for Research and Treatment of Cancer also recommend the use of CSFs in patients on regimens associated with a 20% or greater risk of FN (Aapro *et al*. 2006; Smith *et al*. 2006). Where the risk of FN is 10–20%, patient-related factors such as age should be taken into account when deciding whether G-CSFs are required. Using prophylactic G-CSF support, planned chemotherapy doses can be administered on time, more frequently and at the desired intensity, thus optimizing the outcome for the patient (Lyman *et al*. 2002).

The use of pegylation technology has created a new molecule, pegfilgrastim (Neulasta®, Amgen, Inc.), with a significantly improved pharmacokinetic profile compared with daily filgrastim. Pegfilgrastim is produced by the covalent attachment of a 20-kDa polyethylene glycol moiety to the N-terminal methionine residue of filgrastim. Pegylation results in reduced renal clearance of pegfilgrastim compared with filgrastim, leaving neutrophil receptor-mediated clearance as the dominant clearance mechanism (Johnston *et al*. 2000). This self-regulated nature of pegfilgrastim ensures a sustained serum concentration of pegfilgrastim during the period of neutropenia and allows the administration of a single dose of pegfilgrastim per cycle of chemotherapy, in contrast to the requirement for a daily subcutaneous injection of filgrastim (Johnston *et al*. 2000; Green *et al*. 2003).

Clinical trials have shown that a single fixed subcutaneous dose of pegfilgrastim 6 mg or 100 µg/kg is comparable in safety and efficacy to daily injections of filgrastim for reducing the duration of severe CIN following myelosuppressive chemotherapy in patients with cancer (Holmes *et al*. 2002a,b; Green *et al*. 2003).

The introduction of pegfilgrastim into clinical use in Spain in 2003 may have had an impact on the pattern of use of daily G-CSFs – whether used for prophylaxis or for treatment – and the CIN-related outcomes of chemotherapy patients. Here, we report the results of a Spanish study that compared the usage patterns of daily G-CSF and pegfilgrastim, and the respective outcomes in adults with non-myeloid malignancies receiving myelosuppressive chemotherapy.

## PATIENTS AND METHODS

The LEARN Study was a multi-centre, retrospective, observational study of patients with non-myeloid tumours who underwent cytotoxic chemotherapy supported by G-CSF treatment. Consecutive medical records from patients with documented treatment with either daily G-CSF or pegfilgrastim over the same time period were obtained from 10 Spanish centres during 2003. The study was approved by the Ethics Committees at all participating centres.

From the medical records obtained, the following data were recorded for each patient on standard data collection forms: demographic details; diagnosis and classification of their cancer; previous radiotherapy and chemotherapy treatment; current radiotherapy and chemotherapy treatment; haematological toxicity associated with chemotherapy (neutropenia); usage pattern of G-CSF [proactive (primary prophylaxis) versus reactive (secondary prophylaxis/treatment) ]; and the incidence of bone pain and other adverse effects relating to the G-CSF used.

The outcome measures assessed were: the proportion of patients with proactive versus reactive use of G-CSFs; the duration of treatment with daily G-CSF; and the incidence of CIN-related outcomes. The CIN-related outcomes measured were: delay or reduction in dose (>3 days delay with respect to planned date of administration; <85% of planned dose administered); incidence of FN; incidence of hospitalization; and antibiotic use (type, dose and duration).

Statistical analysis was descriptive in nature. Categorical end points are summarized by the number and percentage of individuals in each category. Continuous end points are summarized by means and standard deviations. Two-sided 95% confidence intervals are presented where appropriate.

## RESULTS

### Baseline characteristics

Records from a total of 248 patients with documented pegfilgrastim or daily G-CSF use during their chemotherapy were included in the study. A total of 75 (30%) patients received pegfilgrastim only, and 111 (45%) patients received daily G-CSF only (99 received filgrastim and 12 received lenograstim). A total of 62 (25%) patients received both daily G-CSF and pegfilgrastim. These patients were treated in the immediate period following the introduction of pegfilgrastim in Spain when physicians were unfamiliar with using a long-acting G-CSF. Due to the heterogeneity of their management, these 62 patients were excluded from analyses of G-CSF usage and chemotherapy-related complications.

The demographic characteristics and cancer diagnoses of the patients included in the study are shown in Table 1. There were no observed differences in demographic characteristics between the treatment groups. Overall, the most common tumour types were lung (25% of all patients), breast (20%) and malignant lymphomas (20%).

**Table 1:** The demographic characteristics and most common tumour diagnoses of patients included in the LEARN study

Demographic characteristic	Daily G-CSF (*n* = 111)	Pegfilgrastim (*n* = 75)	Both (*n* = 62)
Male [*n* (%)]	60 (54.1)	28 (37.3)	30 (48.4)
Female [*n* (%)]	51 (45.9)	47 (62.7)	32 (51.6)
Age (years) (mean ± SD)	55.4 ± 14.5	57.0 ± 14.8	59.3 ± 15.6
Weight (kg) (mean ± SD)	71.7 ± 13.8	67.0 ± 13.3	68.3 ± 10.9
Tumour type [*n* (%)]			
Breast	18 (16.2)	20 (26.7)	12 (19.4)
Lung	30 (27.0)	17 (22.7)	16 (25.8)
Non-Hodgkin's lymphoma	15 (13.5)	7 (9.3)	14 (22.6)
Hodgkin's lymphoma	7 (6.3)	1 (1.3)	6 (9.7)
Multiple myeloma	3 (2.7)	6 (8.0)	1 (1.6)
Gastrointestinal	14 (12.6)	3 (4.0)	6 (9.7)
Gynaecological	8 (7.2)	10 (13.3)	2 (3.2)
Other	16 (14.4)	11 (14.7)	5 (8.1)

G-CSF, granulocyte colony-stimulating factor; SD, standard deviation.

The previous chemotherapy and radiotherapy treatments of patients included in the study are shown in Table 2. The majority of patients in each treatment group had previously received first-line chemotherapy or were currently receiving first-line chemotherapy. Current chemotherapy regimens for the most common tumour types (breast, lung, non-Hodgkin's lymphoma, Hodgkin's lymphoma and myeloma) are shown in Table 3.

**Table 3:** Current chemotherapy regimens administered to patients with the most common tumour types

Tumour type	Current chemotherapy treatment	Daily G-CSF [*n* (%)]	Pegfilgrastim [*n* (%)]	Both [*n* (%)]
Breast		18	20	12
	Anthracycline-based combination regimens	4 (22.2)	13 (65.0)	8 (66.7)
	Taxane-based combination regimens	11 (61.1)	4 (20.0)	4 (33.3)
	CMF	1 (5.6)	0 (0)	0 (0)
	Others	2 (11.1)	3 (15.0)	0 (0)
Lung		30	17	16
	Platinum-based combination regimens	18 (60.0)	14 (82.4)	10 (62.5)
	Platin + etoposide	8 (26.7)	11 (64.7)	4 (25.0)
	Platin + gemcitabine	4 (13.3)	2 (11.8)	3 (18.8)
	Platin + vinorelbine	4 (13.3)	0 (0)	3 (18.8)
	Platin–taxane combination regimens	6 (20.0)	0 (0)	4 (25.0)
	Taxane-based combination regimens	5 (16.7)	1 (5.9)	1 (6.3)
	Others	1 (3.3)	2 (11.8)	1 (6.3)
Non-Hodgkin's lymphoma		15	7	14
	CHOP 14 + rituximab	4 (26.7)	2 (28.6)	2 (14.3)
	CHOP 21 + rituximab	5 (33.3)	1 (14.3)	6 (42.9)
	Others	6 (40.0)	4 (57.1)	6 (42.9)
Hodgkin's lymphoma		7	1	6
	ABVD	6 (85.7)	1 (100.0)	3 (50.0)
	Others	1 (14.3)	0 (0)	3 (50.0)
Myeloma		3	6	1
	VBMCP/VBAD	2 (66.7)	2 (33.3)	0 (0)
	Melphalan + prednisolone	1 (33.3)	3 (50.0)	0 (0)
	Others	0	1 (16.7)	1 (100.0)

CHOP, cyclophosphamide, doxorubicin, prednisolone, vincristine; CHOP-14, CHOP given on a 14-day cycle; CHOP-21, CHOP given on a 21-day cycle; ABVD, doxorubicin, bleomycin, vinblastine, dacarbazine; VBMCP, vincristine, carmustine, melphalan, cyclophosphamide, prednisolone; VBAD, vincristine carmustine, doxorubicin, dexamethasone; G-CSF, granulocyte colony-stimulating factor; CMF, cyclophosphamide, methotrexate, fluorouracil.

**Table 2:** Previous and current chemotherapy and radiotherapy treatments of patients included in the LEARN study

	Daily G-CSF (*n* = 111)	Pegfilgrastim (*n* = 75)	Both (*n* = 62)
Previous treatment [*n* (%)]			
Previous chemotherapy			
1st line	24 (21.6)	24 (32.0)	4 (6.5)
≥2 lines	9 (8.1)	13 (17.3)	6 (9.7)
Previous radiotherapy	19 (17.1)	20 (26.7)	4 (6.5)
Current treatment [*n* (%)]			
Current chemotherapy			
1st line	90 (81.1)	54 (72.0)	48 (77.4)
≥2 lines	20 (18.0)	21 (28.0)	14 (22.6)
Missing	1 (0.9)		
Current radiotherapy	27 (24.3)	14 (18.7)	14 (22.6)

G-CSF, granulocyte colony-stimulating factor.

### Pattern of G-CSF use

The median number of cycles of chemotherapy per patient was the same in both the daily G-CSF- and pegfilgrastim-treated patients [4 (range 1–13); 4 (range 1–16) respectively]. Similarly, the median number of cycles of chemotherapy in which patients received G-CSF was also the same in both the daily G-CSF- and pegfilgrastim-treated patients [2 (range 1–8); 2 (range 1–7) respectively].

The pattern of G-CSF use among the patients in the study is shown in Table 4. The percentage of patients receiving primary or secondary prophylaxis was comparable between the pegfilgrastim and daily G-CSF groups. However, a potential trend was observed towards less frequent use as treatment in the pegfilgrastim group (17%) compared with the daily G-CSF group (30%).

**Table 4:** The patterns of use of pegfilgrastim or daily G-CSF in cancer patients receiving myelosuppressive chemotherapy

G-CSF usage	Daily G-CSF (*n* = 111)	Pegfilgrastim (*n* = 75)
Primary prophylaxis [*n* (%)]	44 (39.6)	29 (38.7)
Median number of injections per cycle (Minimum, maximum)	6 (1, 13)	1 (1, 3)
Secondary prophylaxis [*n* (%)]	53 (47.8)	36 (48.0)
Median number of injections per cycle (Minimum, maximum)	5 (1, 11)	1 (1, 1)
Treatment [*n* (%)]	33 (29.7)	13 (17.3)
Median number of injections per cycle (Minimum, maximum)	5 (1, 11)	1 (1, 1)

G-CSF, granulocyte colony-stimulating factor.

In the daily G-CSF group, the median number of injections per cycle was comparable, irrespective of whether daily G-CSF was used as primary prophylaxis (6 injections; range 1–13), secondary prophylaxis (5 injections; range 1–11) or treatment (5 injections; range 1–11).

### CIN-related complications

The incidence of chemotherapy-related complications in cancer patients receiving either pegfilgrastim or daily G-CSF is shown in Table 5. Patients who were treated with pegfilgrastim appeared to have a numerically lower incidence of dose reduction due to neutropenia, FN, hospitalization due to FN and antibiotic administration than those who received daily G-CSF. However, due to the descriptive nature of the analysis in this study, a conclusion of the significance cannot be made.

**Table 5:** The incidence of chemotherapy-related complications in cancer patients receiving either pegfilgrastim or daily G-CSF

Chemotherapy-related complications [*n*, % of patients (95% CI) ]	Daily G-CSF (*n* = 111)	Pegfilgrastim (*n* = 75)
Dose delay	51, 46.0 (36.0; 55.0)	33, 44.0 (33.0; 55.0)
Dose reduction	23, 20.7 (14.2; 29.2)	11, 14.7 (8.2; 24.6)
Dose reduction due to neutropenia	23, 20.7 (14.1; 29.2)	5, 6.7 (2.5; 15.0)
Febrile neutropenia	27, 24.3 (17.2; 33.1)	8, 10.7 (5.3; 19.9)
Hospitalization due to febrile neutropenia	22, 19.8 (13.4; 28.3)	7, 9.3 (4.3; 18.3)
Antibiotic consumption due to febrile neutropenia	19, 17.1 (11.2; 25.3)	6, 8.0 (3.4; 16.7)

CI, confidence interval; G-CSF, granulocyte colony-stimulating factor.

### Adverse reactions due to G-CSF therapy

A low incidence of adverse reactions considered by the investigator to be due to G-CSF treatment was observed. Three patients (2.7%) in the daily G-CSF group experienced bone pain compared with one (1.3%) in the pegfilgrastim-treated group. Treatment-related adverse reactions other than pain were reported in six patients (5.4%) and one patient (1.3%) respectively.

## DISCUSSION

In this study of everyday clinical practice in Spain, use of daily G-CSFs and pegfilgrastim followed a similar pattern with respect to primary and secondary prophylaxis, but there was a potential trend towards less frequent use of pegfilgrastim as treatment for FN or CIN. Chemotherapy-induced neutropenia-related complications, including FN, appeared to be less frequent in patients receiving pegfilgrastim than in those receiving daily G-CSFs.

Granulocyte colony-stimulating factor prophylaxis reduces the risk of developing CIN, thereby reducing associated morbidity and mortality. Reducing CIN also has the potential to reduce hospitalization and treatment costs, as well as reducing the requirement for dose reductions or treatment delays, which may themselves compromise clinical outcomes (Aapro *et al*. 2006; Smith *et al*. 2006). As previously mentioned, new guidelines on the use of G-CSFs recommend primary prophylaxis from the first cycle of chemotherapy for all patients with an overall risk of developing FN of 20% or higher (Aapro *et al*. 2006; Smith *et al*. 2006). Secondary G-CSF prophylaxis may be given to patients who have had a prior episode of FN in order to prevent subsequent neutropenic complications.

As indicated by the new guidelines, the efficacy of prophylactic G-CSFs for reducing the incidence, duration and intensity of neutropenia is well established with a variety of chemotherapeutic regimens and tumour types (Crawford *et al*. 1991, 2005; Trillet-Lenoir *et al*. 1993; Green *et al*. 2003;Lyman 2005). Data from individual randomized clinical trials (RCTs) have been supported by results from meta-analyses, which have also demonstrated that G-CSF use reduces the incidence and duration of FN-related hospitalization (Holmes *et al*. 2002b), as well as the need for antibiotic treatment and the risk of infection-related mortality (Kuderer *et al*. 2007).

In the largest double-blind, randomized, placebo-controlled multi-centre study of prophylactic pegfilgrastim support reported to date, women with breast cancer receiving docetaxel chemotherapy (a regimen associated with an expected FN incidence of 20%) were randomized to receive either placebo (*n* = 465) or pegfilgrastim (*n* = 463) administered 24 h after chemotherapy (Vogel *et al*. 2005). The placebo group had an overall incidence of FN of 17% compared with 1% in the pegfilgrastim group. In addition, in the pegfilgrastim and placebo groups, respectively, the incidence of hospitalization was 1% versus 14%, and the use of intravenous antibiotics was 10% versus 2% (*P* < 0.001 in each case). Several large comparative RCTs have demonstrated the equivalent efficacy and safety of pegfilgrastim and filgrastim. In two pivotal trials with a similar design, a single fixed dose of pegfilgrastim [6 mg (*n* = 77) or 100 µg/kg (*n* = 149) ] was compared with daily filgrastim (5 µg/kg/day; *n* = 222) as prophylactic G-CSF support in a total of 467 women with breast cancer receiving doxorubicin and docetaxel chemotherapy. The incidences of FN with filgrastim were 20 and 18% in the two studies compared with 13 and 9% for patients receiving pegfilgrastim (Holmes *et al*. 2002a; Green *et al*. 2003). Indeed, the pooling of data from these two trials in a combined analysis suggests that the risk of FN was significantly lower for pegfilgrastim compared with filgrastim (relative risk = 0.56) (Siena *et al*. 2003).

Clinical studies in patients with solid tumours and lymphomas have shown that a median of 11 injections of daily filgrastim per cycle is required to achieve a reduction in the incidence of grade 4 neutropenia equivalent to that associated with a single injection of pegfilgrastim (Holmes *et al*. 2002a; Green *et al*. 2003; Siena *et al*. 2003; Vose *et al*. 2003). A median of 16 injections may be required in patients with acute myeloid leukaemia (Bosi *et al*. 2004; Sierra *et al*. 2008). In clinical practice, however, there is a tendency to reduce the number of days of G-CSF given per cycle (e.g. to 5 or 6 days). The timing and duration of G-CSF administration following chemotherapy has significant effects on haematological recovery and on the incidence of infections. Studies have shown that FN and infections are more likely to occur during chemotherapy cycles that use a reduced number of days of G-CSF prophylaxis (Crawford *et al*. 1997; Koumakis *et al*. 1999; Kloess *et al*. 2003; Mucenski & Shogan 2003; Scott *et al*. 2003). For example, in an analysis of data from a large breast cancer study, pegfilgrastim was compared with current practice use of G-CSF on days 5–10 of each cycle. The incidence of FN was 7% among patients receiving pegfilgrastim compared with 18% for current practice G-CSF use (Von Minckwitz *et al*. 2008). In the present LEARN study, the median number of injections of G-CSF per cycle was also 5–6. Here, too, there was an indication of superior protection from CIN with pegfilgrastim than with current practice G-CSF use; however, as this was a non-randomized study, this finding must be interpreted with caution.

In addition to its beneficial impact on health outcomes, data suggest that pegfilgrastim is a cost-effective treatment. Based on the findings of pivotal trials, a health economic model from Spain has shown that primary prophylaxis with pegfilgrastim was more cost-effective than 11 days of filgrastim treatment in breast cancer patients (Mayordomo *et al*. 2006).

The trend in the current study towards less frequent use of pegfilgrastim as treatment for CIN is unsurprising since physicians were likely to prefer the flexibility of daily dosing in this setting, especially if FN occurred late in the cycle when use of a long-acting dose of pegfilgrastim would potentially overlap with a subsequent chemotherapy cycle. In any case, the routine therapeutic use of G-CSFs is not recommended (Aapro *et al*. 2006).

In summary, LEARN is the first study to compare patterns of pegfilgrastim and G-CSF use in clinical practice in Spain. Our data reveal that soon after its introduction, pegfilgrastim was being administered as primary and secondary prophylaxis in similar proportions of patients and in similar numbers of cycles to daily G-CSF when used for this purpose. As expected, uptake of pegfilgrastim for the treatment of neutropenia was less marked. Importantly, this study indicates that daily G-CSF prophylaxis was given for only around 5–6 days per cycle in many patients, possibly compromising protection against CIN and related events. Our findings support those of other authors, suggesting that pegfilgrastim given once per cycle may be more efficacious than daily G-CSF administered according to current practice. Such findings would need to be confirmed in further, prospective studies.
